# *Spiroplasma* Are Protective Heritable Symbionts With Low Physiological Impact in the Drosophilid Fly *Zaprionus kolodkinae*

**DOI:** 10.1111/1758-2229.70365

**Published:** 2026-05-28

**Authors:** Nuha Alamer, Stefanos Siozios, Chris Corbin, Emily A. Hornett, Jordan E. Jones, Gregory D. D. Hurst

**Affiliations:** ^1^ Institute of Infection, Veterinary and Ecological Sciences University of Liverpool Liverpool UK; ^2^ Deutsches Zentrum für Integrative Biodiversitätsforschung (iDiv), Martin‐Luther‐Universität Halle‐Wittenberg Office Leipzig Germany

## Abstract

*Spiroplasma* bacteria are widespread associates of insects, with *Drosophila* serving as a key model for understanding maternally inherited symbioses. Most research has focused on the *poulsonii–citri* clade of Spiroplasma, leaving other lineages comparatively understudied. Here, we characterise the symbiosis between the drosophilid *Zaprionus kolodkinae* and its *ixodetis* group *Spiroplasma* (*s*Zko). We assembled a complete genome for *s*Zko, which encodes multiple candidate symbiosis factors, including ankyrin repeat domain proteins and diverse ribosome‐inactivating protein (RIP) toxins typically linked to protective interactions. Notably, the genome also harbours a gene with a predicted ricin B lectin‐binding domain, a candidate for establishing microbe‐insect interactions at the eukaryotic cell surface. Phenotypic assays confirmed maternal inheritance of *s*Zko with no evidence of reproductive parasitic phenotypes. Infected flies were protected against attack by the generalist parasitoid wasp *Leptopilina heterotoma*. There was no detectable impact of *s*Zko on its host's starvation tolerance, suggesting minimal physiological cost to the host, and this low impact was mirrored for the protective symbiont *s*Hy1 in *D. hydei* but contrasted with previous results for *D. melanogaster* . We conclude the *Z. kolodkinae–Spiroplasma* association is primarily defensive, and genomic analysis raises the possibility that protection involves a novel coupling between lectin‐binding domains and RIPs.

## Graphical abstract

*Zaprionus kolodkinae* flies carry a maternally inherited ixodetis clade *Spiroplasma* that protects its host against wasp attack but has low overall physiological impact. Genome analysis presented a set of known symbiosis‐relevant effectors, and one encoding—a ricin B domain protein—that is novel to this *Spiroplasma* genome.

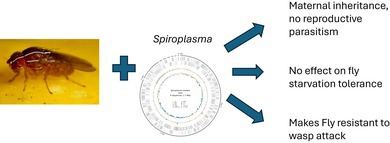

## 1 Introduction

A majority of insect species are infected with heritable microbes—bacteria, viruses and fungi that pass from a female host to her progeny (Duron et al. 2008; Gibson and Hunter 2010; Parker and Rozo‐Lopez 2025). These microbes are important components of insect biology, both through phenotypes that are beneficial to their host, and through reproductive parasitic phenotypes derived from their exclusively maternal inheritance (Hurst 2017). For instance, symbionts are key determinants of host anabolic capacity, desiccation tolerance, and defence against natural enemies (Feldhaar 2011; Engl et al. 2018; King 2019). They also act as antagonists, for instance through male‐killing activity, feminisation of their hosts, and through inducing incompatibility between infected males and females that are either uninfected or carry a different strain of infection (Hurst and Frost 2015). These impacts on individual biology then drive insect ecology and evolution, as antagonists driving the evolution of sex determination systems and as enablers of diversification (Hornett et al. 2022; Cornwallis et al. 2023).

*Spiroplasma* bacteria are widespread associates of insects (Duron et al. 2008). Historically, the genus was one characterised as hemipteran (bug) vectored plant pathogens, such as *S. kunkellii*, *S. phoenecium* and *S. citri* , and as insect pathogens, such as *S. apis* (Gasparich 2010). Increasingly, *Spiroplasma* have become recognised heritable symbionts of insects. The most studied of these is *S. poulsonii* , the causative agent of male‐killing in *D. melanogaster* and willistoni group flies (Williamson et al. 1999; Montenegro et al. 2005). This *Spiroplasma* has additionally been observed to be protective against wasp attack in *D. melanogaster* , with closely related strains in *D. hydei* lacking male‐killing but retaining the protective phenotype (Xie et al. 2010, 2014). Other poulsonii strains have been revealed as protective of flies against nematode attack (Jaenike et al. 2010), and of Tsetse against trypanosomes (Schneider et al. 2019).

The ixodetis clade of *Spiroplasma* is evolutionary distant from the other clades, and contrasts from them in that all members of the clade described to date are heritable. First described from the tick, *Ixodes scapularis* (Tully et al. 1995), the clade is now known to present in diverse arthropods. Members of the clade have been described as protective symbionts of aphids against fungal attack (Scarborough et al. 2005). Male‐killing has also been described as a phenotype of *Spiroplasma* in lepidopterans, aphids and ladybird beetles (Hurst et al. 1999; Jiggins et al. 2000; Simon et al. 2011; Tsugeno et al. 2017), and as causing cytoplasmic incompatibility in a parasitic wasp (Pollmann et al. 2022). To date, only the type species, *S. ixodetis* strain Y32 has been cultured (Tully et al. 1995), and a closed genome sequence subsequently resolved (GCA_030316605.1). Their poor culturability means there are very few complete (closed circle) genomes available compared to other clades (Moore and Ballinger 2023; Arai et al. 2022, 2024).

*Spiroplasma* interactions with flies have received much attention, as it is in this group that mechanistic understanding of their phenotypes has been most commonly obtained. The *Spiroplasma* protein *Sp*AID has been shown to be necessary and sufficient for male‐killing in *D. melanogaster* , and the sex specificity of mortality is identified as deriving from a functional dosage compensation complex (only present in males) that allows *Sp*AID to access the males' single X chromosome (Veneti et al. 2005; Harumoto and Lemaitre 2018). Ribosomal inhibitor proteins (RIP) underpin protective symbiosis, with this toxin impacting eukaryotic ribosome function (Hamilton et al. 2016). Specificity against the worm/wasp enemy is thought to be a consequence of differential internalisation of the RIP protein into enemy vs. host fly cells (Moore and Ballinger 2025). Past work indicated that *S. poulsonii* MRSRO altered metabolism and reduced starvation tolerance in *D. melanogaster* , mirroring observations of physiological impact in the Tsetse fly, *Glossina* (Herren et al. 2014; Son et al. 2021).

Whilst *Drosophila* has proven highly useful as a tool for understanding the mechanism of *Spiroplasma*‐insect interactions, insights have been focussed to a narrow range of strains of *Spiroplasma* in a few *Drosophila* species. Most significantly, there are three well‐studied model systems, *D. melanogaster* , *D. hydei* and *D. neotestacea*, all of which involve the interaction of poulsonii group *Spiroplasma*. This narrow focus contrasts with the widespread incidence of *Spiroplasma*—in around a third of all drosophilid species—and diverse *Spiroplasma* strains present (Watts et al. 2009; Haselkorn et al. 2009). For most of these interactions, we only know that a symbiont exists, with limited information about phenotype. This narrow focus presents a knowledge gap, particularly with respect to using drosophilids to understand the biology of non‐poulsonii clade *Spiroplasma*.

In this paper, we describe the symbiosis between the drosophilid *Zaprionus kolodkinae* and an ixodetis group *Spiroplasma*. The project was initiated by a screen of reads from the 101 genomes project (Kim et al. 2021), which revealed DNA from *Spiroplasma* of the ixodetis group in *Z. kolodkinae* reads (noted also in Moore and Ballinger 2023). This system was of interest as it presented a lab tractable system for the study of ixodetis group *Spiroplasma* in a drosophilid. We assembled a draft genome for the *s*Zko strain from the previously existing read data, obtained the *Z. kolodkinae* fly strain sequenced in the project, and characterised the interaction between host and microbe in terms of inheritance pattern, phenotypic effect on the host in terms of reproductive parasitism and protection, and tested whether carrying *Spiroplasma* was associated with vitality costs or benefits to the host. We conclude this ixodetis *Spiroplasma* is a maternally inherited protective symbiont with low cost of carriage.

## 2 Methods

### 2.1 Genome Assembly and Analysis

#### 2.1.1 Genome Assembly, Annotation and Phylogenetic Analysis

We assembled a genome for *Spiroplasma ixodetis* strain *s*Zko from previously published Oxford Nanopore (ONT) and Illumina datasets (Bioproject PRJNA675888; SRA accessions SRR13070731 and SRR13070681) reported by Kim et al. (2021). More specifically, an initial draft assembly of the ONT dataset was performed using Flye assembler v2.9.6 (Kolmogorov et al. 2019) with the—nano‐hq option. *Spiroplasma* contigs were identified through BLAST searches using the blast package v2.12.0 (Camacho et al. 2009) against a local database consisting of all available complete *Spiroplasma* genomes. ONT and Illumina reads of *Spiroplasma* origin were subsequently extracted by mapping onto the draft assembly using minimap v2.17 (Li 2018) and samtools v1.22 (Danecek et al. 2021). Filtered reads were then used to generate a consensus long‐read assembly using the autocycler pipeline v0.5.2 (Wick et al. 2025). The integrity of the assembly was assessed by mapping the long ONT reads back to it using minimap2 v2.17 (Li 2018) and visually inspecting for misassemblies on igv software v2.19.5 (Robinson et al. 2011). The final assembly was further polished with short reads using one round of polishing with Pilon v1.24 (Walker et al. 2014) and followed by a round of polishing with Polypolish v0.6.1 (Wick and Holt 2022).

The final and polished genome was annotated using BAKTA v1.11.3 with database v6 (Schwengers et al. 2021), and likely gene function informed by additional analysis of protein functional domains using interproscan v5.76–107.0 (Jones et al. 2014). Phage regions were predicted using PHASTEST (PHAge Search Tool with Enhanced Sequence Translation) web server (Wishart et al. 2023). A search for CRISPR‐Cas phage defence systems was performed using the CRISPRCasFinder software (Couvin et al. 2018).

Gene content was then surveyed, and ORFs predicted to encode known features of factors important in symbiosis (ankyrin domains, RIP domains, OTU domains and ETX/MTX2 domains) were examined and manually curated for integrity of the domains and other features of the gene, including the likely presence of a signal peptide. In addition, the predicted gene and domain set was manually examined for other genes related to toxins or virulence.

Core genome phylogenetic analysis was performed on a set of 116 complete *Spiroplasma* genomes. Phylogenetic relationships were estimated on a concatenated set of 62 single‐copy core protein sequences identified as highly conserved mollicute BUSCO v6.0.0 markers (47 when draft genomes included) (Tegenfeldt et al. 2024). To this end, orthologous sequences across the 116 genomes were identified using Orthofinder v3.1.0 (Emms and Kelly 2019), and protein alignments were performed with mafft programme v7.526. Purely aligned regions were removed with the ClipKIT alignment trimming tool v2.6.1 (Steenwyk et al. 2020) under the smart‐gap mode, and the resulting alignments were subsequently concatenated into a super matrix using seqkit v2.8.2 (Shen et al. 2016). Finally, a maximum‐likelihood phylogenetic tree was reconstructed with IQ‐TREE v3.0.1 (Wong et al. 2026) using the best‐fit model, LG + F + I + R6, as identified by ModelFinder (Kalyaanamoorthy et al. 2017).

### 2.2 Inheritance and Testing for Reproductive Parasitic Phenotypes

The infected stock of *Z. kolodiknae* was obtained from Drosophila species centre (https://www.drosophilaspecies.com/shop/d‐kolodkinae/). The lineage is an isofemale line derived originally from Antananarivo in Madagascar, collected in 2008 (Yassin and David 2010). Matched isogenic *s*Zko infected and uninfected lines were obtained through natural segregation, and the curing of the stock confirmed by endpoint PCR over three generations of passage, using primer pair HaIn1 and MGSO that detect a broad range of *Spiroplasma*, including strains from the ixodetis clade (Hurst et al. 1999). DNA quality was confirmed using a QC PCR based on insect mitochondrial COI following Folmer et al. and samples testing negative removed from analyses (primer pair LCO1490: 5′ GGTCAACAAATCATAAAGATATTGG HCO2198: 5′ TAAACTTCAGGGTGACCAAAAAATCA) (Folmer et al. 1994).

We jointly tested mode of inheritance (maternal, paternal and biparental) and presence of sex ratio distortion activity through factorial crosses with *s*Zko infected and uninfected males and females. Seven replicate vials were established for each cross type, and sex ratio was recorded from each vial. F2 progeny were tested for *Spiroplasma* presence by endpoint PCR, alongside QC PCR as above.

In addition, passage of *Spiroplasma* within eggs was confirmed by endpoint PCR on template derived from individual dechorionated eggs from infected and uninfected mothers. Eggs were obtained on grape juice laying plates, the chorion and surface contamination removed through treatment with 2% sodium hypochlorite (bleach) solution for approximately 2–3 min followed by three rinses with sterile water. DNA template was then obtained for individual eggs using Promega wizard protocol (Promega A1125) and tested for *Spiroplasma* presence by endpoint PCR as above, alongside QC PCR.

### 2.3 Capacity to Defend Flies Against *L. heterotoma* Wasp Attack

To determine whether *Spiroplasma ixodetis* confers protection against parasitoid wasps, a wasp attack assay was conducted on *Z. kolodkinae Spiroplasma* infected and uninfected flies. *Z. kolodkinae* is native to Madagascar and no local wasp species/strains were available. We therefore tested protection against *Leptopilina heterotoma* (Madeira strain; Jones and Hurst 2020) a generalist wasp species which has been observed to parasitise and kill flies of the same genus, albeit with low emergence success (e.g., *Zaprionus vittiger*; Schlenke et al. 2007).

To this end, flies were allowed to mate in cages and lay eggs on a grape agar Petri dish painted with live yeast for 48 h. Grape Petri dishes were incubated for a further 48 h to allow larvae to hatch. First instar larvae were picked from the grape plate into the experimental vials at 20 larvae per vial. All experimental vials contained approximately 6 mL of cornmeal agar (1 L water, 10 g agar, 20 g autolysed yeast, 60 g maize flour, 85 g sugar, and 25 mL Nipagin 10% (w/v), Sigma). Four treatments were formed in a 2 × 2 factorial design (*Spiroplasma* status: *s*Zko infected and uninfected × wasp presence: Lh+ and Lh−) with 10 replicate vials/treatment. Five adult female wasps were placed into each vial and allowed to parasitise for 48 h before being removed. An additional control with *Drosophila melanogaster* (Canton‐S strain) was also conducted following the same protocol (wasp presence: Lh+ and Lh−). *D. melanogaster* (Canton‐S strain) has been observed to lack endogenous resistance to *L. heterotoma* (Maderia strain), and this control thus serves to establish wasp potency in a compatible host species. All vials were maintained at 25°C on a 12:12 light:dark cycle. For each vial, the number of pupae, emerging flies, and emerging wasps were recorded. The *Spiroplasma* status of wasp attack survivors was confirmed following eclosion via PCR methods to ensure no contamination of stocks had taken place. The experiment was run in two blocks, separated in time.

Fly survival data were analysed by fitting a generalised linear model with binomial errors and a logit link function. Statistical analyses were conducted in *R* (Version 4.5.2; R Core Development Team 2012) using RStudio. A two‐way factorial analysis of deviance was performed to test the effect of *Spiroplasma* infection and experimental block on fly survival separately in the presence of wasps and in the absence of wasps. The model tested was: glm (fly survival~*Spiroplasma* infection + block), where ‘fly survival’ was a 2‐vector response variable representing the number of surviving flies (successes) and dead flies/pupae (failures) created using the cbind() function in *R*. The ‘Anova’ function in the ‘car’ package version 3.1–3 was used to assess significance using Wald tests (Fox and Weisberg 2019).

### 2.4 Impacts of Spiroplasma on Z. Kolodkinae Vitality, Comparison to *D. hydei*

A starvation tolerance assay was used to assess whether *Spiroplasma* infection influences the overall physiology of *Z. kolodkinae*; costly infections are expected to result in more rapid death on starvation. To this end, 3–5‐day‐old virgin male and female flies from the S^+^ and S^−^ lines were collected and sexed under light CO_2_ anaesthesia. Starvation tolerance was then measured for each sex and infection status groups (*s*Zko infected male, uninfected male, *s*Zko infected female, uninfected female). In each case, five replicates of five flies were placed in a vial containing 1% agar in ddH_2_O to provide moisture while preventing access to nutrients. The vials were sealed with Parafilm to minimise evaporation and maintained at 24°C under standard light–dark conditions. Live/dead status was recorded every 8 h (08:00, 16:00 and 00:00) until all individuals had died.

The recorded survival times (in hours) were used to generate Kaplan–Meier survival curves and estimate median survival for each group. An integrated Cox proportional hazards model was also applied to test the combined effects of infection status (*s*Zko infected vs. unifnected), sex (male vs. female), and their interaction on survival. Survival analyses were performed in R (version 4.4.2; R Core Development Team 2012) using the survival package for Kaplan–Meier and Cox proportional hazards models (Therneau and Lumley 2025) and survminer for visualisation (Kassambara 2023). The model tested was: coxph(Surv(Time_hours, Status)~Sex × Infection). Analyses were run via the R.app GUI on macOS. Visualisations were generated using ggplot2.

Starvation tolerance was likewise analysed in a second symbiosis, *D. hydei* strain TEN104‐102 with and without the poulsonii group symbiont *s*Hy1 (Mateos et al. 2006). The design was different, using a common garden rearing environment and determining *Spiroplasma* infection post hoc by PCR. After ageing up to Day 10, flies were moved with a pooter into individual, 1.5% (w/v) agar‐bottomed plastic vials, which were closed with Parafilm (Bemis Company Ltd.) to stop the agar desiccating. Each fly was given a unique identifier linking it to records of its emergence date, starvation start date, sex, and vial of origin. The starvation vials were stored in the same 25°C incubator, and the trays housing them were turned and rearranged daily. Vials were checked for starvation deaths every 8 h after 36 h. When flies were found dead or no longer capable of standing upright or walking, the hour of the observed death was recorded and the fly was preserved in a 95%‐ethanol‐filled screw‐cap vial for later estimation of infection status. The infection status of each fly was recovered post hoc by PCR assay. Flies were transferred to individual Eppendorf tubes, dried on heat blocks, the DNA extracted with the Promega Wizard extraction kit, and *Spiroplasma* presence tested with PCR assays using primer pair SpoulF/SpoulR (Montenegro et al. 2005) and CO1 primers for extraction efficacy quality control (as detailed above). The infection status of each fly was then paired to its starvation data. Full methodological details can be found in Corbin (2018).

The *D. hydei* survival data were analysed with the function survreg() in the package ‘survival’ (Therneau and Lumley 2025) in R version 3.0.2 (R Core Development Team 2012), using a Weibull accelerated failure time model. The Weibull model was chosen over a Cox model, because analysis using the cox.zph() function demonstrated that the data violated the assumption of proportional hazards. Additionally, the Weibull function permits different scale and shape functions to be fitted to the data by sex, which was necessary as the male and female distributions were different shapes. The maximal model was the survival function in terms of sex, strata(sex) (which tells the model to fit shape and scale parameters separately for each sex), infection, and the interactions. The model was refined using anova() to compare simpler models to the maximal. The final, minimal model included sex and strata(sex), but not infection.

## 3 Results

### 3.1 Genome Assembly and Analysis

The genome of *s*Zko assembled into one circular chromosome of 1.43 MB and seven small circular plasmids (evidenced as closed circles and containing partitioning genes). The total estimated genome size was 1.67 MB (Tables 1 and S1 and Figure S1) and contained an estimated 2180 protein coding genes (Table S2). Phylogenetic analysis placed *s*Zko in the ixodetis clade, within a lineage of phylogenetically closely related strains allied to strains from several hymenopteran, lepidopteran and other dipteran hosts (Figure S2), and related to the ixodetis strain previously characterised in the drosophilid fly *D. atripex*.

**TABLE 1:** Genome description for *s*Zko *Spiroplasma* from *Z. kolodkinae*.

Total assembly size	1,673,605 bp
Size of main chromosome (circular)	1,429,949 bp
Number of plasmids (circular)	7
Number of protein coding genes	2180
Coding density	81.6%
Number of rRNA operons (16S, 23S, 5S)	2
Number of tRNA	27
GC%	23.6%
Number of predicted phages regions (intact/questionable/incomplete)	0/1/0
CRISPR Cas	No
Number of genes with RIP domains	10 (chr.)
Number of genes encoding OTU domains	1 (chr.)
Number of genes encoding ETX/MTX2 domains	1 (chr.)
Number of genes with ankyrin repeat domains	10 + 3 pseudogenes

**FIGURE 1:**
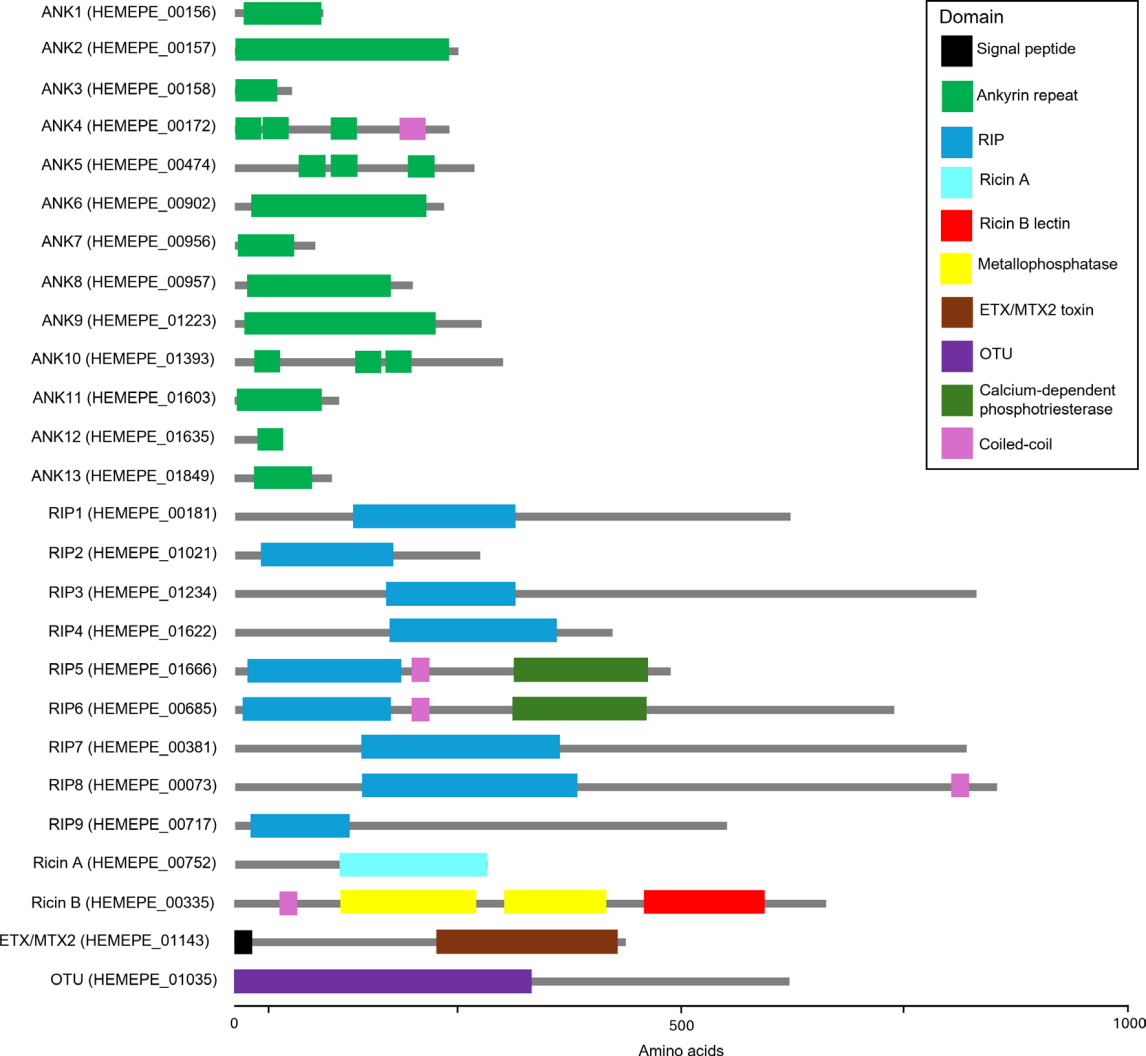
Structure and diversity for proteins in *s*Zko predicted to carry domains of relevance to symbiosis. ANK, ankyrin domain; ETX/MTX2, beta‐pore‐forming toxin domain; OTU, ovarian tumour deubiquitinase domain; RIP, ribosomal‐inactivating protein domain.

The genome sequence did not evidence presence of a CRISPR defence system, and PHASTEST predicted a single putative prophage region, categorised as ‘questionable’ (in terms of completeness). Examining the genome for evidence of factors related to symbiosis via Bakta annotation or interpro domain estimation (Tables S2 and S3) revealed 13 ORFs that carried ankyrin repeat domains (of which three are predicted to pseudogenes), 10 ORFs with predicted RIP/Ricin (A subunit) domains, one gene with an OTU domain and another with an ETX/MTX domain (both toxin domains) (Figure 1). These were all located on the main chromosome. Of these, only a single protein—that containing an ETX/MTX domain—was predicted to have a signal peptide. A search for the two domains previously suggested as candidates for causing CI in *Spiroplasma* (HMG‐domain and PD‐(D/E)XK nuclease domain genes: Pollmann et al. 2022) indicated these were present, with the *s*Zko genome carrying three copies of HMG‐domain containing genes (all on plasmids), and one copy of a gene with a PD‐(D/E)XK nuclease domain (main chromosome).

One additional ORF of note combined a predicted metallophosphatase domain with a ricin B‐like lectin domain. This ORF is of interest as the ricin holotoxin from castor oil plants is a composite of a toxic A chain element with an RIP domain bound by a disulphide bond to a lectin B chain protein (Lord et al. 2003). BLAST search revealed the closest related genes to the *s*Zko ORF were in ixodetis *Spiroplasma*, but protein sequence similarity was restricted to the N terminus metallophosphatase domain with no evidence for the lectin domain found at the C terminus. Interestingly, the lectin‐binding domain at the C terminus most closely matched an effector protein from *Wolbachia w*CauA.

### 3.2 Inheritance and Testing for Reproductive Parasitic Phenotypes

Reciprocal crosses between infected (*s*Zko infected) and uninfected flies produced the pattern expected from maternal only inheritance (Table 2). All offspring from *s*Zko infected females were infected, regardless of the father's infection status (Crosses A and C), while no infection was detected in F2 offspring from uninfected females, even when the male in the cross was infected with *Spiroplasma* (Crosses B and D). Maternal inheritance through eggs was further confirmed by PCR assays for *Spiroplasma* from dechorionated egg template. All 77 eggs from five vials derived from infected female *Z. kolodkinae* tested positive for *Spiroplasma* on PCR assay, whereas none of the 77 eggs from vials derived from uninfected females tested positive.

**TABLE 2:** Results of reciprocal crosses between *s*Zko infected and uninfected *Zaprionus kolodkinae*, showing infection status of F2 flies as determined by endpoint PCR, and sex ratio of F1 flies.

Cross type	Female parent	Male parent	F2 offspring infection status	Replication for transmission	F1 sex ratio
A	*s*Zko infected	Uninfected	All infected	88 flies from seven vials	55 f:33 m
B	uninfected	*s*Zko infected	All uninfected	100 flies from seven vials	94 f:74 m
C	*s*Zko infected	*s*Zko infected	All infected	100 flies from seven vials	59 f:58 m
D	Uninfected	Uninfected	All uninfected	100 flies from seven vials	111 f:104 m

To test whether infection influences offspring sex ratio, the number of males and females emerging from each cross was recorded in the F1 generation (Table 2). There was no evidence of variation in sex ratio between cross types (Chi‐squared test for heterogeneity using summed data within cross types: *χ* ^2^_3 df_ = 1.88, *p* = 0.60). Overall, the proportion of male offspring produced by *s*Zko infected females (cross types A and C) was 0.44 (*N* = 205, binomial CI: 0.36 ≤ *p* ≤ 0.52), while that produced by S^−^ females (cross types B and D) was 0.46 (*N* = 383, binomial CI: 0.41 ≤ *p* ≤ 0.52).

A prediction of symbionts creating cytoplasmic incompatibility is that crosses between uninfected females and infected males perform poorly, with low egg viability. While counting F1 adults is not a formal measurement of CI (which would ideally assess egg hatch rate), crosses of type B (uninfected females × *s*Zko infected males) were healthy, producing more F1 offspring than cross of type C (*s*Zko infected females × *s*Zko infected males), indicating CI either does not exist or is rather weak in this system.

### 3.3 Capacity to Defend Flies Against *L. heterotoma* Wasp Attack

The survival of *Spiroplasma*‐infected and uninfected flies was compared in the presence and absence of *L. heterotoma* attack. The experiment was run in two blocks, separated in time, each alongside a positive control for wasp attack potency using *D. melanogaster* .

*Leptopilina heterotoma* reduced larva‐adult and pupa to adult survival in *Z. kolodkinae* lacking *Spiroplasma* (Table 3). This viability loss on exposure to wasps was not observed when the *Z. kolodkinae* carried *Spiroplasma*. Wasp attack was highly efficient against the control host, uninfected *D. melanogaster* . Statistical analysis provided no evidence for a difference in survival of *s*Zko and uninfected flies in the absence of wasp attack (Effect of *Spiroplasma*: *χ* ^2^ = 0.0002, df = 1, *p* = 0.988; Random effect Block: *χ* ^2^ = 12.6924, df = 1, *p* < 0.001). Contrastingly, differences in survival between infected and uninfected flies were evidenced in the presence of wasps (Spiroplasma: *χ* ^2^ = 25.490, df = 1, *p* < 0.001; Block: *χ* ^2^ = 18.268, df = 1, *p* < 0.001).

**TABLE 3:** Survival of flies to pupation and adulthood for *Z. kolodkinae* with and without Spiroplasma in the presence/absence of *L. heterotoma* wasps, alongside wasp emergence from vials.

Fly	Spiroplasma infection status	Wasp attack status	Larva‐adult survival %	Pupa‐adult survival %	Wasps emerging %	*N*
Block 1						
*Z. kolodkinae*	*s*Zko+	Lh−	59.00	62.63	—	100
*Z. kolodkinae*	*s*Zko+	Lh+	47.00	57.26	1.00	100
*Z. kolodkinae*	Uninfected	Lh−	57.50	62.13	—	40
*Z. kolodkinae*	Uninfected	Lh+	28.33	37.06	3.33	60
*D. melanogaster*	Uninfected	Lh−	84.00	96.5	—	100
*D. melanogaster*	Uninfected	Lh+	5	6	NA	100
Block 2						
*Z. kolodkinae*	*s*Zko+	Lh−	78.00	84.85	—	180
*Z. kolodkinae*	*s*Zko+	Lh+	68.50	77.74	0.50	200
*Z. kolodkinae*	Uninfected	Lh−	75.00	76.73	—	200
*Z. kolodkinae*	Uninfected	Lh+	46.00	53.26	9.50	200
*D. melanogaster*	Uninfected	Lh−	83.00	94.78	—	200
*D. melanogaster*	Uninfected	Lh+	5.50	11.28	42.50	200

*Note:* Survival of CS *D. melanogaster* (*Spiroplasma* uninfected) provided as positive control for wasp attack efficiency/development. Experiment was conducted in two blocks separated by 1 month. NA, not ascertained.

Abbreviation: NA, not ascertained.

Adult wasps were observed to emerge at a low rate from *Z. kolodkinae* without *Spiroplasma*, but only very sporadically where *Z. kolodkinae* was *Spiroplasma* infected (*Z. kolodkinae* without *Spiroplasma*: wasps completed development in 9.5% of larval prey, with *Spiroplasma*: 0.5%; *D. melanogaster* control—42.5%). Statistical analysis rejected the null hypothesis of no effect of *s*Zko on parasite emergence rate from flies (Fisher exact test for wasp emergence rate for *Z. kolodkinae s*Zko infected vs. uninfected: *p* < 0.001).

### 3.4 Impacts of Spiroplasma on *Z. kolodkinae* Vitality, Comparison to *D. hydei*

**FIGURE 2:**
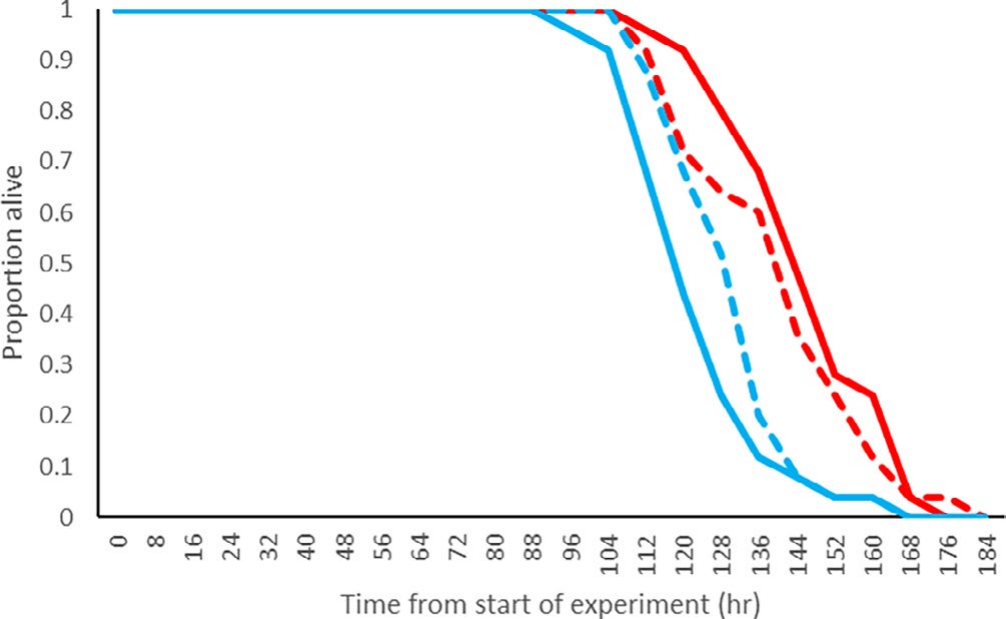
Survival curves for S^+^ and S^−^ males and females of *Zaprionus kolodkinae* under starvation. Blue = male flies, Red = Female flies, Dashed line = *s*Zko infected Solid line = uninfected flies. Sample size = 25 for each category.

Analysis of starvation tolerance indicated male *Z. kolodkinae* had lower starvation tolerance than female, with no overall impact of *Spiroplasma* infection (Figure 2). Median survival times were S^−^ males: 120 h, S^+^ males 136 h, S^−^ females: 144 h, S^+^ females 144 h. The Cox proportional hazards model revealed a significant effect of sex on survival time (*p* < 0.001) but no effect of infection status alone nor any interaction between sex and infection status (*p* = 0.52, *p* = 0.20, respectively). Males exhibited a higher hazard ratio (HR = 4.32), indicating lower survival compared to females.

**FIGURE 3:**
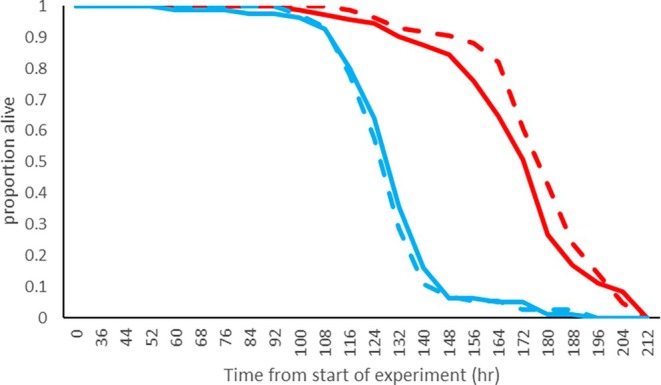
Survival curves for *s*Hy1 and uninfected males and females of *D. hydei* under starvation. Blue = male flies, Red = female flies, Dashed line = *s*Hy1 infected solid line = uninfected flies. Sample size = 25 for each category. Sample sizes: *s*Hy1 infected female *N* = 84; uninfected female *N* = 71; *s*Hy1 infected male *N* = 83; uninfected male *N* = 73.

Likewise, *D. hydei* males survived less long than females in the absence of food, but there was no influence of *Spiroplasma s*Hy1 on survival in either sex (Figure 3). Infection was dropped as a factor during ANOVA model testing, as it made no improvement to the model. Thus, infection does not significantly change the time taken for flies to starve. However, the effect of sex is highly significant (*p* = 2.62 × 10^−70^). For females, the Weibull scale function (which gives an impression of the characteristic lifespan) is 185 h, while for males it is 140 h. This difference reflects the much higher longevity of females compared to males under starvation, which itself is probably the result of larger average body size in females.

## 4 Discussion

This study aimed to characterise the symbiosis between *Spiroplasma s*Zko and the drosophilid fly *Z. kolodkinae*. Our data indicate the ixodetis group *Spiroplasma* is maternally inherited through eggs (high confidence), does not exhibit reproductive parasitism in the form of sex ratio distortion or cytoplasmic incompatibility (high confidence and medium confidence, respectively), but does have the capacity to defend its host against attack by a generalist parasitic wasp (high confidence). Starvation tolerance assays showed no effect of symbiont presence on this metric, indicating physiological impacts are low (medium confidence; limitations of statistical power, and particular assay conditions including CO_2_ anaesthesia). Overall, our characterisation of the symbiosis is one likely maintained through the benefits of protection against natural enemies with low direct costs.

Protective symbiosis was established in our study using a generalist wasp species, *L. heterotoma*. The protection observed is quite marked, with wasp attack having a minimal effect on the survival of *Spiroplasma*‐infected flies, contrasting to 30%–40% mortality where *Spiroplasma* was absent. However, our generalist wasp, while able to parasitise and kill *Z. kolodkinae*, was clearly an inefficient parasite (did not kill all *s*Zko‐uninfected flies, poor wasp survival from these hosts). The importance of the protective trait in nature will depend on the rate of attack by the locally present wasps and the protection afforded against them. The wasp fauna resident on Madagascar includes *Leptopilina orientalis* (boulardi group) and *L. victoriae* (*heterotoma* group) (Allemand et al. 2002). *Leptopilina heterotoma* presence is noted in earlier records that predate DNA barcoding (Quinlan 1988), but is not listed as present in southern Africa by Allemand et al. (2002). Onward work would need to examine protection afforded by *s*Zko against attack by *L. orientalis* and *L. victoriae* to gain understanding of the importance of protection in the field setting.

Our results nevertheless indicate protection of flies against wasp attack by *Spiroplasma* is found broadly across *Spiroplasma* strains. Protective symbiosis was first described *in D. hydei* (vs wasps), *D. neotestacea* (vs nematodes) and *D. melanogaster* (vs wasps) (Xie et al. 2010, 2014; Jaenike et al. 2010). These *Spiroplasma* strains are from the poulsonii clade. Our study is of an ixodetis group *Spiroplasma* and shows defensive capacity extends across the clade. The data complements the observation noted in Moore and Ballinger (2023) that the *Spiroplasma* strain in *D. atripex*, also in the ixodetis group, is also protective, and the previous work showing ixodetis *Spiroplasma* defend aphids against fungal attack (Scarborough et al. 2005). However, two strains in the *citri* group carried by *D. aldrichi* and *D. mojavensis* did not evidence a protective phenotype against wasps despite carrying genes encoding RIP domains (Ramirez et al. 2025). Collectively, these studies indicate defence against natural enemies is a common but not universal phenotype of heritable *Spiroplasma* strains, with some strains additionally showing reproductive parasitic phenotypes and others without any currently described impact on the host.

Members of the ixodetis clade of *Spiroplasma* have now been recorded as having male‐killing capacity (in ladybird, aphid and lepidopteran hosts) (Hurst et al. 1999; Jiggins et al. 2000; Simon et al. 2011; Tsugeno et al. 2017), protection against wasps (in fly hosts—this study) and fungi (in aphid hosts) (Scarborough et al. 2005), and causing CI (in wasp hosts) (Pollmann et al. 2022). However, in many cases the impact of the symbiont on the host is not known. This is particularly true of tick hosts, from which *S. ixodetis* was first isolated, where this heritable symbiont is found in a broad range of species (e.g., Binetruy et al. 2019; Ogata et al. 2021), and which have considerable importance as disease vectors. Ticks are commonly parasitised by encyrtid wasps (Hu et al. 1998; Ramos et al. 2023), and an obvious onward line of research is to examine if the *Spiroplasma* provides protection to the tick host in a similar manner to that seen in flies.

The genome of *s*Zko revealed a diverse range of symbiosis factors. Ixodetis group *Spiroplasma* represent a group of largely heritable microbes that encode a broader range of proteins for interface with the host than other *Spiroplasma*. For *s*Zko this include diverse ankyrin repeat containing proteins, RIP domain contains proteins, one gene with an OTU domain and another with an ETX/MTX domain (the latter three being toxin domains). These domains are widely found in insect associated *Spiroplasma* (Moore and Ballinger 2023). The high number of ankyrin domain proteins is typical of ixodetis group *Spiroplasma*. The ankyrin repeats themselves are generally involved in binding to eukaryotic proteins, but the bioactivity of the protein then depends on other domains present, which were able to identify in only a single case. The 10 genes predicted to encode RIP/ricin domains is higher than other annotated genomes to date, which have from 0 to 8 genes of this type. A notable feature was that the proteins above were in only one case predicted to carry a signal peptide (the ETX/MTX domain protein). This observation implies either that alternate, non sec‐dependent means of secretion exist for these proteins, or that the bioactivity of the proteins does not rely on secretion. In particular, the capacity for *s*Zko to produce protection does not appear to require secretion of RIP‐domain proteins through the sec system.

One gene encoded a symbiosis‐relevant protein that is not present in other sequenced *Spiroplasma* genomes: a protein with a ricin B chain lectin domain. One notable feature of this *s*Zko gene was its chimeric origin. BLAST homology indicated the N terminus of the protein showed similarity to predicted proteins from ixodetis *Spiroplasma* (which did not themselves have the lectin‐binding domain), and the lectin domain at the C terminus hits to *Wolbachia* ORFs. The *s*Zko ORF thus appears to be the product of genetic exchange between symbionts within the intracellular environment, as per the intracellular arena hypothesis (Bordenstein and Wernegreen 2004). However, any exchange is unlikely to be recent, as the *Z. kolodkinae* strain does not itself harbour *Wolbachia*, and the amino acid identity of the domain is quite distinct.

Ricin B chain proteins are utilised in the context of the RIP toxin complex in plant systems but are also more widely deployed in the maintenance of symbiotic interaction. The B chain protein binds to lectins (carbohydrate) at the eukaryotic cell surface. In the ricin toxin complex, the B chain protein is covalently bound via disulphide bridges to the ricin A chain that is the active RIP and enables the entry of the bioactive ribosome‐inhibiting ricin A into the eukaryotic cell (Lord et al. 2003). The lectin domain can also have biological function independently of the RIP/ricin A protein. For instance, microsporidial adherence to host cells and infectivity is mediated by a B chain lectin (Liu et al. 2016; Prybylski et al. 2022) and B chain lectin domain proteins are present in other symbionts (such as *Wolbachia*) that do not carry RIP proteins. The presence of this domain in a genomic background with multiple genes encoding ribosome‐inactivating peptides (that are ricin A analogues) presents the tempting hypothesis that it interacts with RIP proteins in protective symbiosis (in *s*Zko but no other *Spiroplasma*). However, the ricin B chain ORF in *Spiroplasma* was not located alongside genes encoding an RIP that would implicate a holotoxin model.

We also compared the starvation tolerance of *s*Zko infected and uninfected flies but observed no difference. This lack of effect was mirrored for *s*Hy1in *D. hydei* , where we could likewise detect no difference in starvation tolerance between *s*Hy1 infected and uninfected flies. Starvation tolerance captures overall physiological activity—including metabolic rate—and it is expected that a costly symbiont would impact survival under starvation. The absence of an observable effect contrasts with data from *D. melanogaster* —where *s*Mel infected flies survived less long that uninfected comparators on nutrient‐free diets (Herren et al. 2014). It is notable that the two strains in *Drosophila* that are purely protective (*s*Zko, *s*Hy1) have minimal impact on starvation tolerance whereas that which additionally shows male‐killing has been found to be costly. This contrast is consistent with pleiotropic autoreactive costs in females associated with *s*Mel male‐killing behaviour. Future work should examine other impacts of *Spiroplasma* on host physiological performance. Negative Impacts of *Spiroplasma* on fecundity have previously been observed in Tsetse fly (Son et al. 2021), but effects were either absent or very weak in *D. melanogaster* (Montenegro et al. 2006; Herren et al. 2014) and not apparent as single infections in aphids (Łukasik et al. 2013).

## 5 Conclusion

The *s*Zko strain has high maternal inheritance, but with some segregational loss (our uninfected lineage was derived from the parent line following inefficient vertical transmission). A lack of impact on starvation tolerance implies a low metabolic cost, which should be corroborated with study of the impact of the strain on host fecundity. The strain does not exhibit reproductive parasitism but does enable protection of the fly against a generalist wasp. Our study has one key limitation: it is based on a single isofemale line, maintained at the *Drosophila* species centre since its collection in 2008 and our conclusions are thus limited to the focal combination under study. Populations that harbour protective symbionts with imperfect vertical transmission without CI are expected to contain a mix of infected and uninfected individuals. We would predict that field collected *Z. kolodkinae* in Madagascar would likewise have a mix of *s*Zko infected and uninfected flies, and this would then represent natural variation in resistance to natural enemy attack.

## Author Contributions

**Nuha Alamer:** conceptualization, investigation, funding acquisition, writing – original draft, formal analysis, visualization, data curation. **Stefanos Siozios:** investigation, writing – original draft, formal analysis, visualization, data curation. **Chris Corbin:** investigation, writing – original draft, formal analysis. **Emily A. Hornett:** investigation, writing – review and editing, visualization, formal analysis. **Jordan E. Jones:** conceptualization, investigation, writing – original draft, formal analysis, visualization, data curation, supervision. **Gregory D. D. Hurst:** conceptualization, investigation, funding acquisition, writing – original draft, formal analysis, supervision, data curation.

## Funding

This work was supported by the Natural Environment Research Council (NE/V011979/1) and Saudi Arabian Cultural Bureau.

## Ethics Statement

This study was not conducted on animals as defined by the Animals (Scientific Procedures) Act 1986.

## Conflicts of Interest

The authors declare no conflicts of interest.

## Supporting information


**Figure S1:** Genome of *S. ixodetis* strain *s*Zko. Circos plot of Main genome and seven associated plasmids, all linearised, indicating CDS, tRNA, rRNA and GC skew.
**Figure S2:** Phylogenetic placement of Spiroplasma strain *s*Zko (red) in the genus, alongside host/isolation source, based on a concatenated set of 62 single‐copy genes. Phylogenetic tree was estimated using maximum‐likelihood reconstruction with IQ‐TREE v3.0.1 using the best‐fit model, LG + F + I + R6, as identified by ModelFinder.

**Table S1:** emi470365‐sup‐0002‐Tables.xlsx. *Spiroplasma ixodetis* *s*Zko plasmid features.
**Table S2:** Bakta annotation of the *s*Zko genome.
**Table S3:** Domain annotations for the *s*Zko genome.

## Data Availability

The data that support the findings of this study are openly available in zenodo (genome assembly) and NERC EDS Environmental Information Data Centre (underpinning data from experiments) at https://doi.org/10.5281/zenodo.18176388 and https://doi.org/10.5285/f31c08e9‐7feb‐4ccb‐8c83‐304176e1135a.
