# Supplementary material for: Spiroplasma Are Protective Heritable Symbionts With Low Physiological Impact in the Drosophilid Fly Zaprionus kolodkinae
Source: Environ Microbiol Rep. 2026 May 28;18(3):e70365. doi: 10.1111/1758-2229.70365 (PMC13240588; doi:10.1111/1758-2229.70365)
Supplement: Supplementary file 1 — Figure S1: Genome of S. ixodetis strain sZko. Circos plot of Main genome and seven associated plasmids, all linearised, indicating CDS, tRNA, rRNA and GC skew. Figure S2: Phylogenetic placement of Spiroplasma strain sZko (red) in the genus, alongside host/isolation source, based on a concatenated set of 62 single‐copy genes. Phylogenetic tree was estimated using maximum‐likelihood reconstruction with IQ‐TREE v3.0.1 using the best‐fit model, LG + F + I + R6, as identified by ModelFinder. [file EMI4-18-e70365-s001.docx]

Supplementary figures


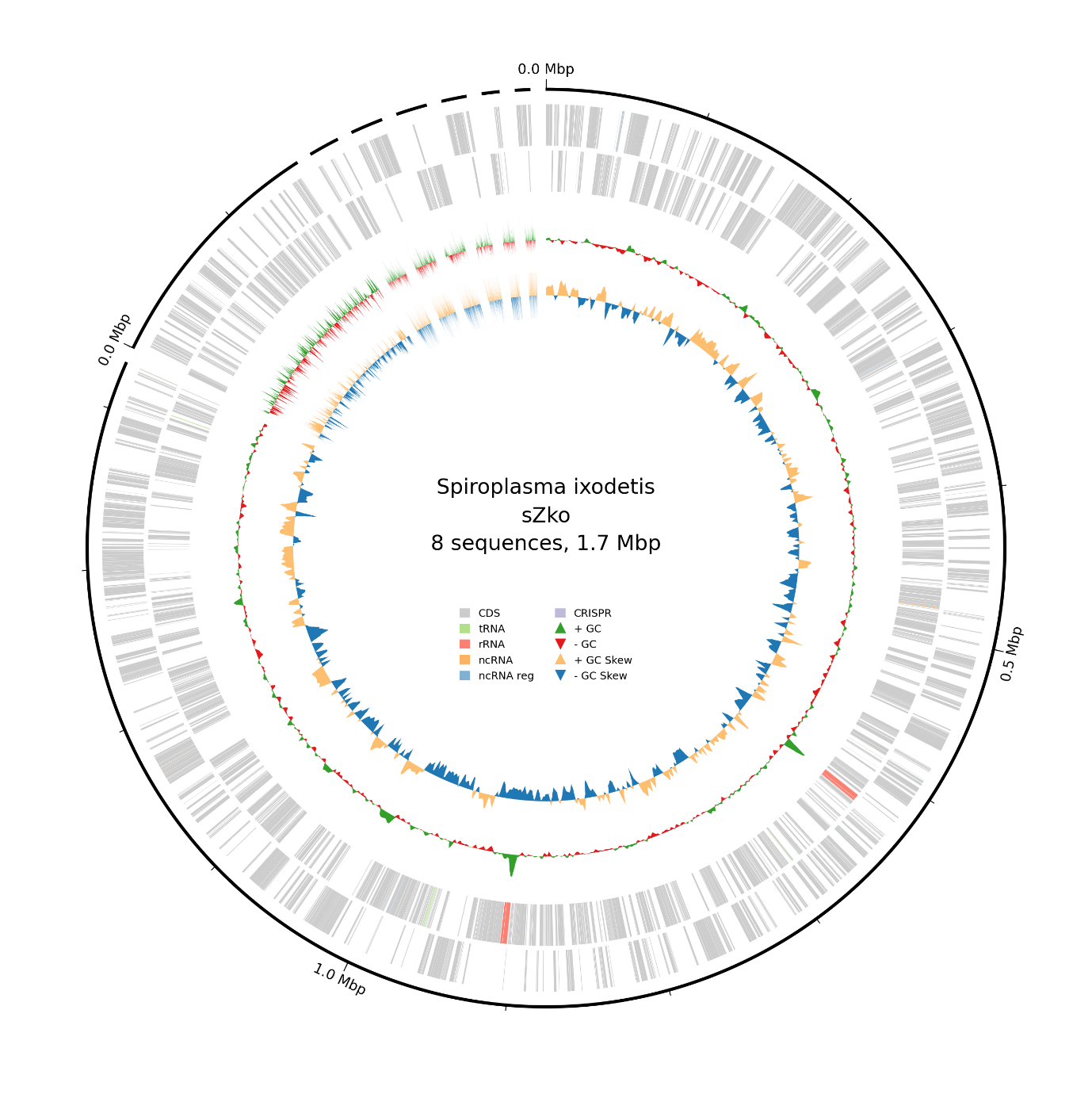


Figure S1: Genome of *S. ixodetis* strain sZko. Circos plot of Main genome and 7 associated plasmids, all linearised, indicating CDS, tRNA, rRNA and GC skew.


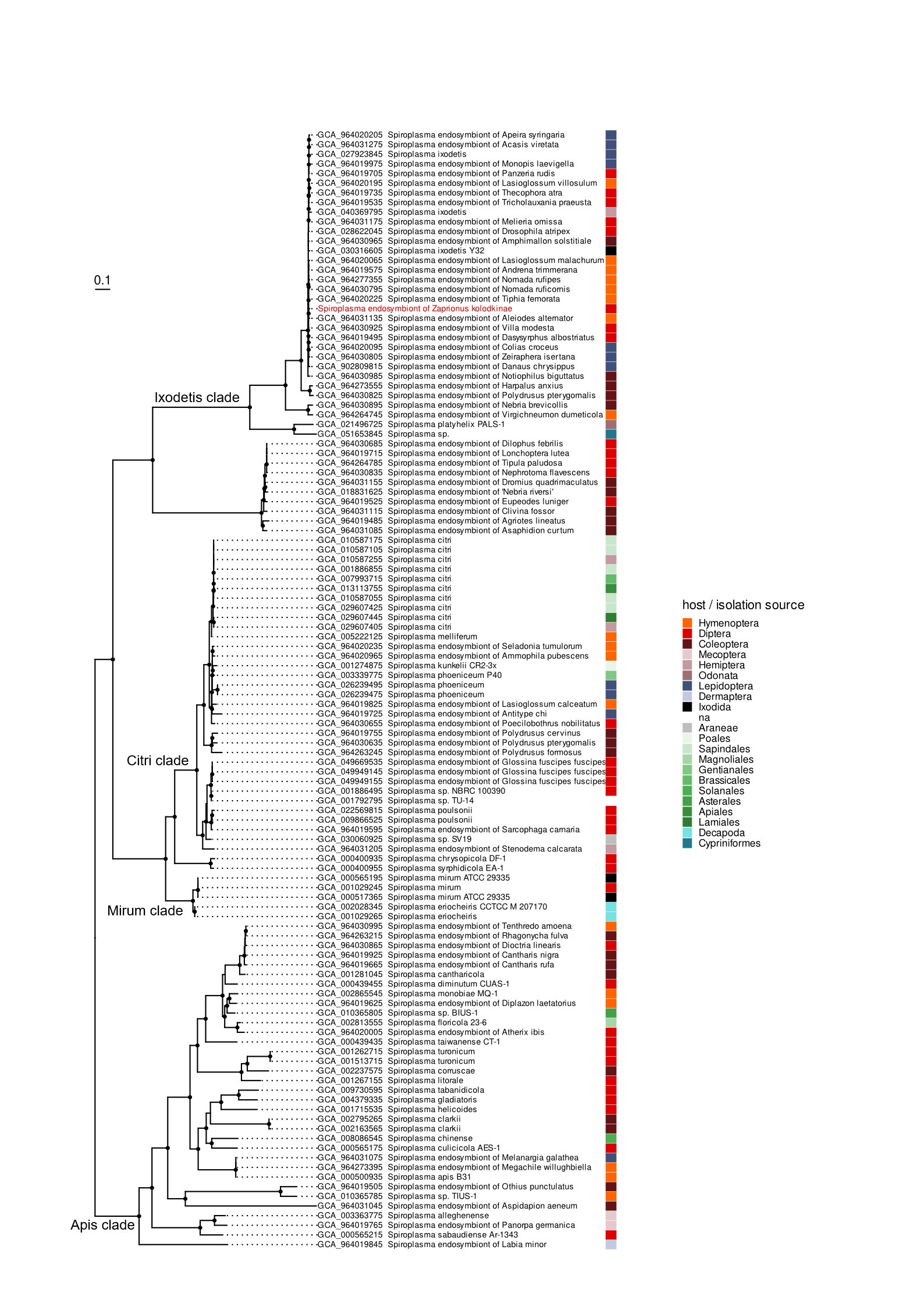


Figure S2: Phylogenetic placement of Spiroplasma strain *s*Zko (red) in the genus, alongside host/isolation source, based on a concatenated set of 62 single-copy genes. Phylogenetic tree was estimated using maximum-likelihood reconstruction with IQ-TREE v3.0.1 using the best-fit model, LG+F+I+R6, as identified by ModelFinder
